# NF-kappaB Mediated Transcriptional Repression of Acid Modifying Hormone Gastrin

**DOI:** 10.1371/journal.pone.0073409

**Published:** 2013-08-23

**Authors:** Dipanjana Datta De, Arindam Datta, Sumana Bhattacharjya, Susanta Roychoudhury

**Affiliations:** Cancer Biology and Inflammatory Disorder Division, CSIR-Indian Institute of Chemical Biology, Kolkata, India; Aarhus University, Denmark

## Abstract

*Helicobacter pylori* is a major pathogen associated with the development of gastroduodenal diseases. It has been reported that *H. pylori* induced pro-inflammatory cytokine IL1B is one of the various modulators of acid secretion in the gut. Earlier we reported that IL1B-activated NFkB down-regulates gastrin, the major hormonal regulator of acid secretion. In this study, the probable pathway by which IL1B induces NFkB and affects gastrin expression has been elucidated. IL1B-treated AGS cells showed nine-fold activation of MyD88 followed by phosphorylation of TAK1 within 15 min of IL1B treatment. Furthermore, it was observed that activated TAK1 significantly up-regulates the NFkB subunits p50 and p65. Ectopic expression of NFkB p65 in AGS cells resulted in about nine-fold transcriptional repression of gastrin both in the presence and absence of IL1B. The S536A mutant of NFkB p65 is significantly less effective in repressing gastrin. These observations show that a functional NFkB p65 is important for IL1B-mediated repression of gastrin. ChIP assays revealed the presence of HDAC1 and NFkB p65 along with NCoR on the gastrin promoter. Thus, the study provides mechanistic insight into the IL1B-mediated gastrin repression via NFkB.

## Introduction

About half of the world’s population harbors the gastric pathogen *Helicobacter pylori* [1]. An interesting feature of *H. pylori* mediated gastro-duodenal infection is the development of a wide spectrum of diseases in infected individuals. While a majority of the infected individuals remain asymptomatic, a fair proportion develop gastritis, some of whom further progress to either gastric ulcer, carcinoma or low grade B-cell lymphoma [2]. While duodenal ulcer is characterized by increased acid output, gastric cancer is associated with lower acid secretion [3,4]. Gastrin is one of the major acid regulators in the gut [4]. It is known that in response to an infection, the gastric mucosa elicits an inflammatory response characterized by the release of cytokines like IL1B, TNF-α etc [5]. In this context, we previously described that IL1B polymorphism plays an important role in *H. pylori* mediated duodenal ulcer and it inhibits gastrin synthesis via Smad7 and NFkB pathway [6,7].

The exact mechanism by which IL1B down-regulates gastrin through NFkB remains unknown. In the absence of appropriate stimuli, the NFkB dimer (homodimer or heterodimers) is sequestered by the IkB complex in the cytoplasm [8,9]. Upon stimulation, an upstream signal, transduced primarily by kinases (ERK/Protein kinases/ MAPK/JNK/p38/PI3K), phosphorylates the IkB complex, leading to its degradation by the ubiquitination pathway. This results in release of the sequestered NFkB and its translocation to the nucleus to affect gene transcription [10]. However, this well established model of NFkB activation is not complete as reports suggest the presence of NFkB in the nucleus of non-stimulated cells [10–12]. These findings raise questions regarding the basal transcriptional machinery of certain gene promoters involving NFkB [12–15]. Moreover, the upstream signaling molecules that release inactive NFkB from inhibitory IkB complex also modify it by phosphorylation/acetylation at specific residues [16–18]. Such modifications work as an imprint for NFkB to further associate with co-regulators and ultimately affect gene transcription [16–18]. There are many reports that suggest association of certain co-regulators with specific modifications on NFkB [19]. In this context, it is important to note the emerging role of NFkB in transcriptional repression [11–13], although it is primarily considered a transcriptional activator [20]. Many studies have reported that NFkB homodimers serve as transcriptional repressors, justifying that these homodimers lack the transactivation domain and bring about gene repression. However, there are a few recent reports that document NFkB heterodimers down-regulating gene expression [11–13].

In this study we delineate the possible IL1B responsive signaling pathway that activates NFkB and affects gastrin expression. We further describe the role of the NFkB heterodimer as a transcriptional repressor of gastrin expression in the presence of IL1B, and also suggest its potential to positively regulate the basal activity of the gastrin promoter. We propose that NFkB can associate with different co-factors producing opposing effects on the gastrin promoter.

## Materials and Methods

### Cell culture, transfection and treatment with IL1B, TSA, NBD and siRNA

Human gastric cancer cell line AGS (CRL-1739,ATCC,USA) was maintained in RPMI 1640 medium (Invitrogen, Carlsbad, CA, USA) supplemented with 10% fetal calf serum (Invitrogen, Carlsbad, CA, USA) and antibiotics (1% Pen Strep and 0.006% Gentamicin, Invitrogen, Carlsbad, CA, USA) at 37^0^C in 5% CO_2_ incubator (Innova Co-170, New Brunswick Scientific, India). AGS cells plated in 35mm dish (0.5×10^6^) were transiently transfected in duplicates with different amounts of DNA as required for the specific experiment using Lipofectamine 2000 Reagent (Invitrogen, Life Technologies, Carlsbad, USA) according to the manufacturer’s protocol. PGL3 control vector (Promega, Madison, USA) was used as control for luciferase assay. To account for variable transfection efficiency between samples, cells were co-transfected with the β-gal expressing reporter plasmid pSV- β -Galactosidase Control Vector (E-1081, Promega, Madison, USA). AGS cells were treated either with varying amounts (0–10 ng/ml) of recombinant IL1B (Sigma Aldrich, St. Louis, USA) for two and a half hr or with 10ng/ml recombinant IL1B for different time points. Twenty-four hr after transfection, AGS cells were incubated with 150 µM of nemo binding inhibitory peptide (NBD) (Calbiochem-Novabiochem, San Diego, USA) for additional 24 hr and harvested for luciferase assay. AGS cells transfected with 240 Gas-Luc were treated with Trichostatin A (Calbiochem-Novabiochem, SanDiego, CA), a histone deactylase inhibitor, at 100 nM for 48 hr and harvested for further experiments. The siRNA directed against TAK1 (Santa Cruz Biotechnology, CA), p300 (Ambion, Life Technologies, Foster City, USA) and control scrambled siRNA (Ambion, Life Technologies, Foster City, USA) were used at a final concentration of 80 nM for 48 hr. All treatments except the transfections were performed at 10% serum containing medium.

### Quantitative Real Time PCR

Total cellular RNA was extracted from recombinant IL1B treated AGS cells by Trizol method according to the manufacturer’s protocol (Invitrogen, Life Technologies, Carlsbad, USA). cDNA was prepared using random hexamer (Invitrogen, Life Technologies, Carlsbad, USA) with MMLV RT (Promega, Madison, USA). Relative mRNA expression was determined by real time RT PCR in the ABI 7500 Fast or StepOne (Applied Bio-systems Inc, Life Technologies, Foster City, USA) using the SYBR Green technology (Applied Biosystems Inc, Life Technologies, Foster City, USA). The primer pairs used are as follows: MyD88 forward(F) 5’-CGGCAACTGGAGACACAAG-3’ and MyD88 reverse (R) 5’-TCTGGAAGTCACATTCCTTGC-3’ *;* Beta-actin forward (F) 5′-GGATGCAGAAGGAGATCACTG-3′ and Beta-actin reverse (R) 5′-CGATCCA CACGGAGTACTTG-3′; TAK1 forward(F) 5′-CAGAGCAACTCTGCCACCAGTA-3 ′and TAK1 reverse (R) 5′-CATTTGTGGCAGGAACTTGCTCC-3′; p300 forward(F) 5′-TAAACTCTCATCTCCGGCCC-3 ′and p300 reverse (R) 5′-CCACCATTGGTTAGTCCCAA-3′; 18srRNA forward (F) 5′-TGACTCTAGATAACCTCGGG-3′ and 18srRNAreverse (R) 5′-GACTCATTCCAATTACAGGG-3′. Threshold cycle C_T_ of duplicate samples was determined using the ABI 7500 Fast or StepOne software (Applied Biosystems Inc, Life Technologies, Foster City, USA). The expression of target genes were normalized to Beta–actin or 18srRNA levels by calculating the ΔC_T_ value which is defined as C_T_ (threshold cycle) of the endogenous control subtracted from the C_T_ of the target genes. The fold differences of target gene expression were calculated using the formula 2 ^–ΔΔC^_T._

### Western Blot analysis

IL1B treated AGS cells (2.0×10^6^ cells) were subjected to western blot analysis using antibodies against TAK1(1:500 dilution, #4505, Cell Signaling, USA), p-TAK1(1:100 dilution, #4536, Cell Signaling, USA), TAB1(1:500 dilution, #3226, Cell Signaling, USA) NFkB p50 antibody (1:500 dilution, #3035, Cell Signaling, USA), NFkB p65 antibody(1:500 dilution, Sc-7151,Santa Cruz Biotechnology Inc, Santa Cruz, USA and #6956, Cell Signaling, USA), NFkB phosphop65S536 antibody(1:100 dilution, Santa Cruz Biotechnology Inc, Santa Cruz, USA), or Beta -actin (Sigma Aldrich, St. Louis, USA). The immune complexes were detected by staining with HRP-conjugated secondary antibody (Sigma Aldrich, St. Louis, USA). The band intensities were quantified by using ImageJ (http://rsb.info.nih.gov/ij/index.html). The integrated density of each band was normalized to the corresponding human Beta-actin band.

### Luciferase and β-galactosidase Assay

The cells were lysed in the luciferase cell culture lysis buffer provided with the Luciferase Assay Kit (Promega, Madison, USA) and 15 µl of supernatant was analyzed for firefly luciferase activity. Luminescence was measured as relative light units (RLU), taking the reading of luciferase assay substrate alone and then with lysate in GlowMax 20/20 luminometer (Promega, Madison, USA). The total protein concentration in each lysate was determined by a protein assay kit (Sigma Aldrich, St. Louis, USA) and subsequently used to normalize the luciferase activity. Transfection normalization was done by beta galactosidase assay. β-gal assay was performed using β-gal assay kit according to the manufacturer’s protocol (Promega, Madison, USA). Luciferase activity was normalized with the corresponding β-gal activity and expressed as relative light unit/µg of protein. Measurements of mean ± S.D of relative luciferase unit per microgram of protein were taken in triplicates and represented graphically as mean ±S.D on a MS-Excel sheet.

### Co-immunoprecipiation Assay

Recombinant IL1B (10ng/ml) treated and untreated AGS cells were washed with PBS and the pellet was resuspended in 200 mM NaCl, 20 mM Tris-HCl (pH 7.5), 1% Triton X-100, 1 mM DTT, and Protease Inhibitor Cocktail (Sigma Aldrich, St. Louis, USA). The cells were lysed by freeze-thaw cycle and the supernatant was treated overnight with either anti-HDAC1 or anti-p300 antibody (Santacruz Biotechhnology, Santacruz, USA). Rabbit normal IgG (Sigma Aldrich, St. Loius, USA) was taken as a control for immunoprecipitation. The antibody protein complex was precipitated with Protein-G sepharose beads (Genei, Bangalore, India), washed and eluted by SDS-lysis buffer. The eluted sample was then processed for western blot analysis with anti NFkB p65 antibody.

### Electrophoretic Mobility Shift Assay (EMSA)

IL1B treated AGS cells (8.2 X 10^6^ cells) were harvested in 1ml of ice-cold PBS and washed with hypotonic buffer (10mM Hepes pH 7.9, 1.5mM MgCl_2_, 10mM KCl, 0.5mM PMSF and 0.5mM DTT) by centrifuging at 10,000 rpm for 5 min at 4^o^C. Washed cells were resuspended in hypotonic buffer containing 0.5% NP-40 and centrifuged at same speed. The nuclear pellet thus obtained was lysed in lysis buffer (20mM Hepes pH 7.9, 420mM NaCl, 1.5mM MgCl_2_, 0.2mM EDTA, 25% glycerol, 0.5mM PMSF) and centrifuged at 14,000 rpm for 10min at 4^0^C. The supernatant nuclear extract was mixed with storage buffer (10mM Hepes pH 7.9, 50mM KCl, 0.2mM EDTA, 20%(v/v) glycerol, 0.5mM PMSF, and 0.5mM DTT) and kept at -80^o^C for further use. Protein concentrations of the nuclear extracts were determined by Bradford method (Sigma- Aldrich, St.Loius, USA). The oligos (at equal concentration) of the putative NFkB site on gastrin promoter are sense: 5’-TGACCCCCAGGATATGGTGGG-3’ and antisense: 5’-ACTGGGGGTCCTATACCACCC-3’ [NT_086877, GeneID: 2520] and that of consensus NFkB binding sites are sense: 5’-AGTTGAGGGGACTTTCCCAGGC-3’ and antisense: 5’-GCCTGGGAAAGTCCCCTCAACT-3’. The annealed oilgos were labeled with (γ-^32^P) dATP using T4 polynucleotide kinase (Promega, Madison, USA). Gel supershift assay was performed by incubating the nuclear lysates prepared from AGS cells either with NFkB p50 (ab7971, Abcam, Cambridge,USA) or NFkB p65 antibody (ab7970, Abcam, Cambridge, USA) for 30 minutes followed by addition of the radio-labelled gastrin promoter probe. After incubation the samples were immediately loaded on to a 5% nondenaturing polyacrylamide gel and run at 200 V for 2 hr. Gel was dried and exposed to X-ray film.

### Chromatin Immunoprecipitation Assay (ChIP)

Chromatin immunoprecipitation (ChIP) assay was performed according to the protocols for Imgenex Quick Chip Kit (Imgenex, CA, USA). AGS cells were either treated with IL1B (10ng/ml) or left untreated and harvested after two and a half hr. Cells were resuspended in lysis buffer and sonication was performed to obtain 200-1000bp DNA fragments. The sheared DNA was then cleared off debris and immunoprecipitation was performed at 4°C overnight with anti NFkB p50 (ab7971, Abcam, Cambridge,USA), anti NFkB p65 (ab7970,Abcam, Cambridge,USA), anti NCoR (ab24552,Abcam, Cambridge, USA), anti HDAC1 (Santa Cruz Biotechnology Inc. Santa Cruz, USA), anti H3K9Me3 (ab8898, Abcam, Cambridge, USA), and anti p-300 antibody (N15, sc584, Santa Cruz Biotechnology Inc, Santa Cruz, USA). Subsequently the immunoprecipitated DNA was assayed by quantitative Real Time PCR using Gastrin forward (F) 5’-AAGCATTGCTCCTGACCCAG-3’, Gastrin reverse (R) 5’- ACCCTGCCATATGAGTCCAG-3’ primer pairs in ABI StepOne (Applied Bio-systems Inc, Life Technologies, Foster City, USA) with SyBR Green (Applied Bio-systems Inc, Life Technologies, Foster City, USA) technology for promoter occupancy. Primers against GAPDH promoter (GAPDH forward: 5’-AAAAGCGGGGAGAAAGTAGG-3’, GAPDH reverse: 5’-AAGAAGATGCGGCTGACTGT-3’) were used as negative control. Data was plotted in MS Excel as a percentage of input from mean of three different experiments performed.

### ELISA

AGS cells (0.5X10^6^) were plated in 35mm dish and either treated with IL1B (10ng/ml) or left untreated for 2 hr. The cell lysates were prepared and protein estimation was done by Bradford reagent (Sigma Aldrich, St. Louis, USA) as per the manufacturer’s protocol. Equal amount of protein lysates were analyzed for gastrin (gastrin-17 polypeptide) protein using Gastrin Immunoassay kit (Assay Design Inc, Ann Arbor, USA) according to the manufacturer’s protocol.

## Results

### IL1B activates NFkB through TAK1 to inhibit gastrin expression

**Figure 1:**
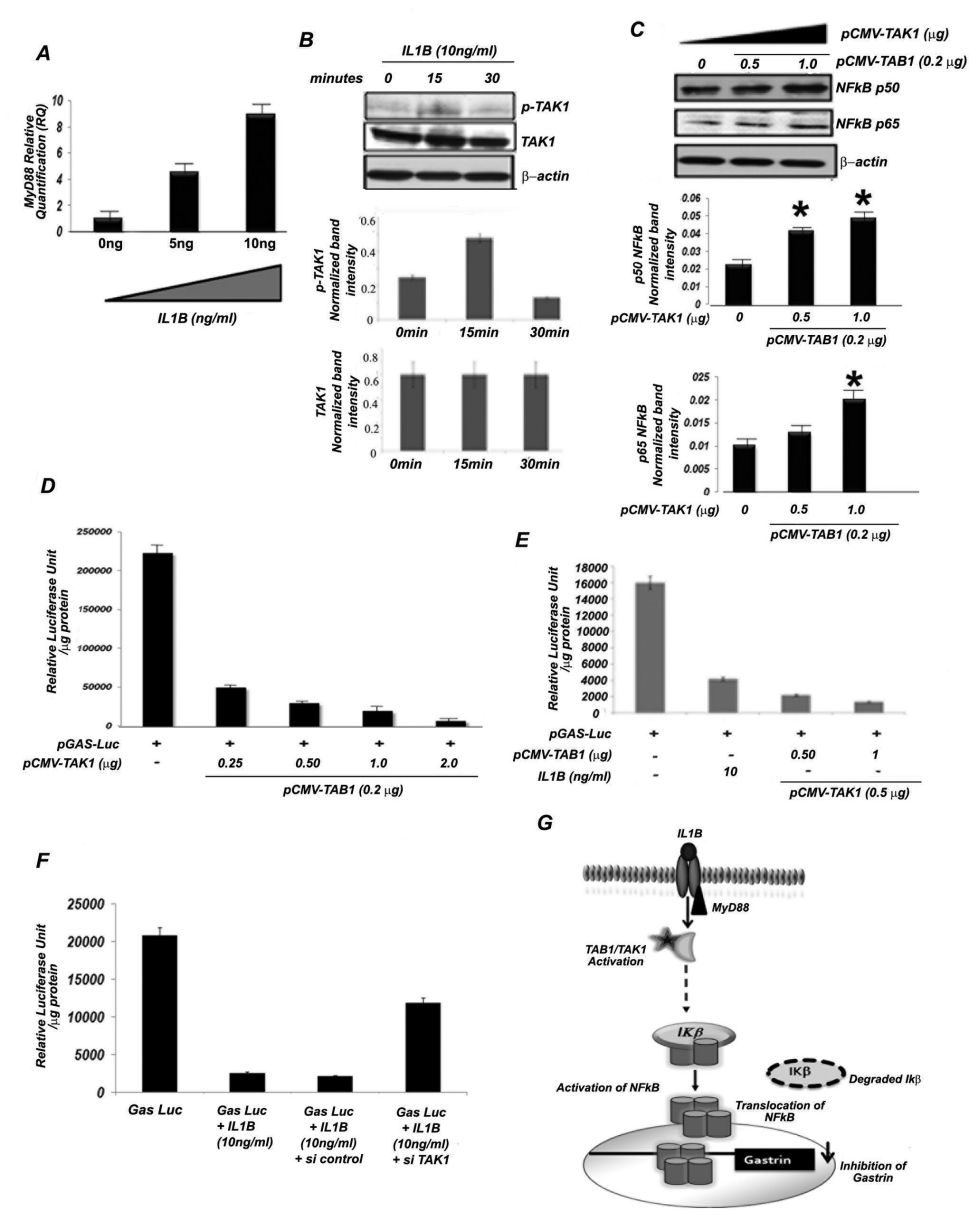
IL1B-induced TAK1 activates NFkB to inhibit gastrin expression. (A) Dose dependent activation of MyD88 by IL1B in AGS cells. Total RNA was extracted from AGS cells treated with increasing concentration of recombinant IL1B (0, 5, 10ng/ml) for 10 min. Real time PCR analysis for MyD88 was performed in those RNA samples. β-actin was taken as the endogenous control. The graph represents the mean of relative quantification measured from three different experiments +/- SD. (B) Analysis of IL1B induced phosphorylation of MAP3K TAK1. AGS cells were treated with 10ng/ml recombinant IL1B protein for 0, 15 and 30 min and then lysed for western blot analysis with p-TAK1, TAK1 and β–actin antibodies. A representative blot is shown. The band intensities were scanned by imageJ and the normalized mean band intensities of three independent experiments with +/-SD values graphically plotted. (C) Dose dependent activation of NFkB p50 and p65 by TAK1. AGS cells were co-transfected in a dose dependent manner with pCMVTAK1 along with its activator pCMVTAB1 and western blot analysis was done for NFkB p50 and p65 respectively. A representative blot is shown. The band intensities were scanned by imageJ and the normalized mean band intensities of three independent experiments with +/-SD values graphically plotted. (D and E) Effect of TAK1/TAB1on gastrin promoter activity. AGS cells were co-transfected with gastrin luciferase (pGAS-Luc) and either with (D) varying concentrations of pCMVTAK1 along with 0.2 µg of pCMVTAB1 or (E) varying concentrations of pCMVTAB1 along with 0.5 µg of pCMVTAK1.The IL1B (10ng/ml) treatment as control has been included in panel E. Cells were harvested after 48 hr of transfection for luciferase assay. The normalized mean Relative Luciferase Unit/µg protein +/- SD of three different experiments was plotted. (F) Knock down of TAK1 in IL1B-treated AGS cells releases gastrin repression. AGS cells were first transfected with either TAK1 siRNA (80 nM) or control siRNA(80 nM). After twenty four hr these cells were transfected with 0.5 µg of pGAS-Luc.Forty six hr post pGAS-Luc transfection, cells were treated with IL1B (10ng/ml) for two hr and subsequently harvested. Control experiments with only pGAS-Luc transfected and IL1B treated pGAS-Luc transfected AGS cells are also shown. (G) A cartoon showing that IL1B induces NFkB via MyD88/TAK1 to regulate gastrin expression. Stars in panel C indicate statistical significance of the observations.

Chakravorty et al. previously established that IL1B-stimulated expression of NFkB leads to down-regulation of the gastrin promoter, indicating NFkB as a repressor of gastrin transcription [6]. However, the specific pathway by which IL1B induces NFkB and down-regulates gastrin remains largely unknown. IL1B recognizes its cognate receptor IL1R, and initiates a cascade of signaling events that involves different adaptor proteins. Myeloid differentiation factor 88 (MyD88) is one such widely utilized adaptor molecule in IL1R signaling [21]. We observed a dose dependent increase in MyD88 mRNA in AGS cells upon treatment with IL1B for 15 min (Figure 1A). MyD88 subsequently interacts with other receptor associated protein kinases that ultimately impinge on TAK1 (transforming growth factor β-activated kinase 1) and activates the transcription factor NFkB (p50/p65) [22]. However, MyD88 dependent activation of NFkB can also be induced by TAK1 independent pathways, as reported in TAK1 deficient murine embryonic fibroblasts, where incomplete suppression of IL1R or LPS-induced NFkB activation was observed [23]. We had reported that pharmacological inhibitors of possible signaling pathways, like PD-98059 for ERK and SB-202190 for p38 MAPK, do not have significant effects on gastrin repression by IL1B [6]. It was also not found to be significantly affected by kinase inhibitors like phosphokinase A inhibitor (H89), phoshotyrosine kinase inhibitor (Genestin), phospholipase C inhibitor (Neomycin Sulphate), phosphokinase C inhibitor (Staurosporine) [6]. Thus, we investigated the role of TAK1 in IL1B mediated activation of NFkB in AGS cells. AGS cells were treated with 10ng/ml IL1B and harvested at different time points (as described in Figure 1B) to detect phosphorylated TAK1 by western blot analysis. As stimulation by recombinant IL1B is transient, phosphorylation of TAK1 peaked at 15 min of treatment and the signal reduced at 30 min (Figure 1B). There was no change in the level of total TAK1 protein upon IL1B treatment (Figure 1B).

We next investigated whether IL1B-activated TAK1 is able to induce NFkB and inhibit gastrin expression. AGS cells were co-transfected with increasing doses of pCMVTAK1 and pCMVTAB1. TAB1, a mammalian TAK1 adaptor protein, interacts constitutively with TAK1 and induces TAK1 kinase activity when over-expressed [24]. We observed ~4 fold (Figure 1C, lower panel and histogram) and ~5 fold (Figure 1C upper panel and histogram) activation of p65 and p50, respectively, at the highest concentration of transfected pCMVTAK1 (Figure 1C). Overexpression of pCMVTAK1 and pCMVTAB1 has been shown in Figure S1A. To validate that activated TAK1 was indeed the molecular intermediate between IL1B and NFkB for gastrin repression, AGS cells were co-transfected with pGas-Luc, pCMVTAK1 and pCMVTAB1, and luciferase assay was performed after 48 hr of transfection. We observed that increasing concentrations of either pCMVTAK1 (Figure 1D) or pCMVTAB1 (Figure 1E) repressed gastrin promoter activity in a dose-dependent manner. To ensure that TAK1 was involved, AGS cells were co-transfected with pGas-Luc and siRNA against TAK1 for 46 hr, treated with recombinant IL1B (10ng/ml) for 2 hr, and a luciferase assay was performed. We found that knock down of TAK1 by specific siRNA was able to alleviate the down-regulation of gastrin by IL1B (Figure 1F and Figure S1B), supporting the role of TAK1 in regulation of the gastrin promoter. Thus, these experiments indicate that IL1B induced TAK1 activates NFkB (p50/p65) to down- regulate gastrin (Figure 1G).

### IL1B-dependent post-translational modifications of NFkB p65 influence gastrin repression in AGS cells

**Figure 2:**
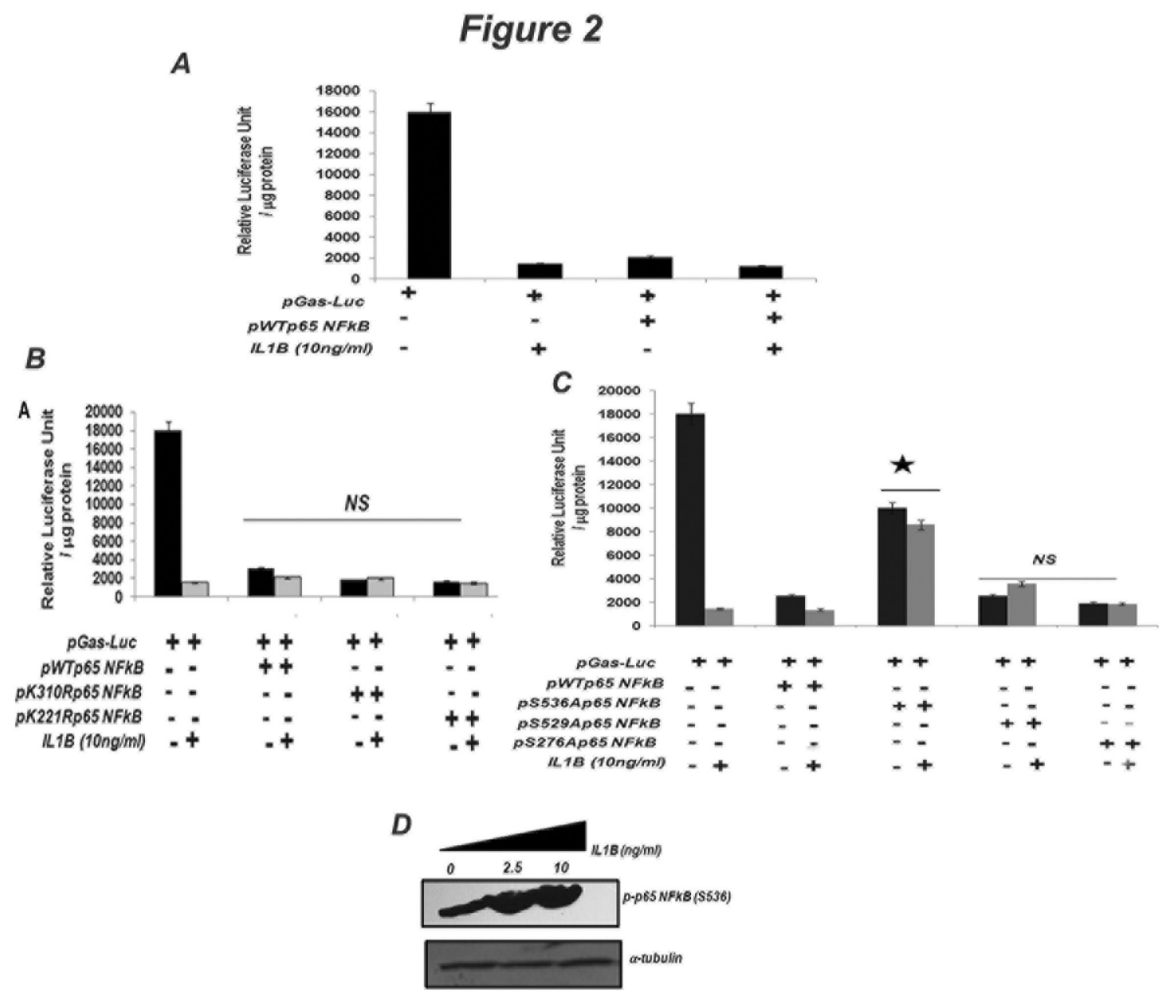
IL1B represses gastrin promoter by inducing S536 phosphorylation of NFkB p65. (A) Effect of NFkB p65 on gastrin promoter activity. AGS cells were co-transfected with gastrin luciferase (pGAS-Luc) along with 0.2µg of NFkB p65 (pWTp65NFkB). Forty six hr after transfection, cells were either treated or left untreated with 10ng/ml of IL1B for two hr and then harvested for luciferase assay. The normalized mean Relative Luciferase Unit/µg protein +/- SD of three different experiments was plotted. (B and C) Effect of NFkB p65 mutants on gastrin promoter activity. AGS cells were co-transfected with gastrin luciferase (pGAS-Luc) along with (B) either NFkB p65 (pWTp65NFkB) or the lysine NFkB p65 mutants (pK310R p65 NFkB and pK221R p65 NFkB) and (C) with either NFkB p65 (pWTp65NFkB) or with the serine NFkB p65 mutants (pS536A p65 NFkB, pS529A p65 NFkB and pS276A p65 NFkB). Forty six hr after transfection, cells were either treated or left untreated with 10 ng/ml of IL1B for two hr and then harvested for luciferase assay. 0.2µg of wild type or mutant NFkB p65 were used for transfection. The normalized mean Relative Luciferase Unit/µg protein +/- SD of three different experiments was plotted. (D) Analysis of IL1B induced phosphorylation of NFkB p65 at ser536 residue. AGS cells were treated with varying concentrations of recombinant IL1B protein for two hr and then lysed and immunoblotted with anti p-p65NFkB (S536) and anti α-tubulin antibodies. A representative blot is shown. The star in panel C indicates that the repression of transcription of pGAS-Luc with pWTp65NFkB is significantly alleviated when p65S536ANFkB was used both in presence and absence of IL1B.NS indicates that the differences between the repressive activity of pWTp65NFkB and that of the mutant clones on gastrin promoter are not significant.

Activation of NFkB involves modifications (phosphorylation, acetylation, sumoylation and ubiquitination) on specific residues that determine both the strength and duration of the NFkB-mediated transcriptional response [25]. We observed that pWTp65 NFkB, when transfected in AGS cells, was able to repress gastrin expression both in the presence and absence of IL1B (Figure 2A). To further dissect the mechanism of NFkB p65-mediated gastrin repression, the effects of various NFkB p65 mutants were studied (Figure 2B and Figure 2C). The transcriptional repression activity of various lysine mutants of NFkB p65 (K310R p65 NFkB, K221R p65 NFkB) remained unchanged both in the presence and absence of IL1B on the pGas-Luc promoter, suggesting that acetylation at these sites is not involved in NFkB-mediated inhibition of gastrin expression (Figure 2B). However, the transcriptional repression activity of the S536A mutant of NFkB p65 on the pGas-Luc promoter was significantly abrogated both in the presence and absence of IL1B in AGS cells (Figure 2C). In contrast, the inhibitory activities of pS276Ap65 NFkB and pS529Ap65 NFkB were comparable to that of pWTp65 NFkB suggesting that these sites are not involved in the transcriptional repression of gastrin (Figure 2C). There was ~1.8 fold repression of gastrin luciferase in presence of pS536Ap65 NFkB in contrast to 9 fold repression by pWTp65 NFkB (Figure 2C). We also show IL1B-induced specific phosphorylation at serine 536 residue of wild type NFkB p65 in a dose dependent manner (Figure 2D). These results suggest that IL1B- induced S536 phosphorylation of NFkB is responsible for transcriptional repression of gastrin expression.

### NFkB along with HDAC1 negatively influences the gastrin promoter in AGS cells

**Figure 3:**
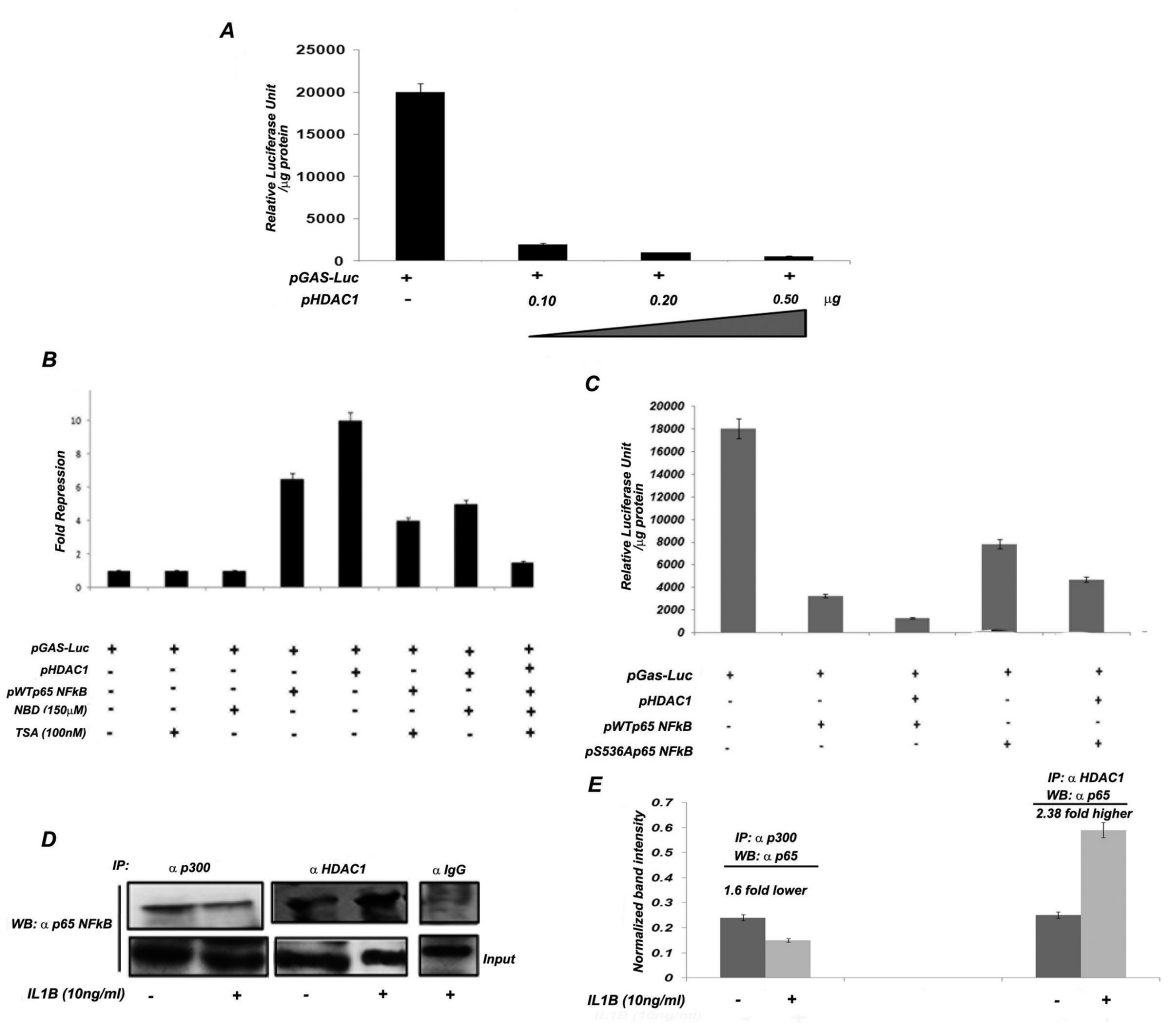
HDAC1 associates with NFkB to repress gastrin promoter activity. (A) Effect of HDAC1 on gastrin promoter activity. AGS cells were co-transfected with pGAS-Luc construct and increasing amount of HDAC1 expression vector. Cells were harvested 48 hr after transfection and luciferase activity was measured. The mean luciferase activity of three different experiments +/- SD is graphically plotted. (B and C) Combined effect of NFkB p65 and HDAC1 on gastrin promoter activity. (B) AGS cells were co-transfected with pGAS-Luc construct along with pHDAC1 or pWTp65NFkB or both. Transfected cells were then incubated with either NBD (150µM) for 24 hr or TSA (100nM) for 48 hr or both or left untreated. Cells were harvested after 48 hr of transfection. The mean luciferase activity of three different experiments is represented as fold repression with respect to gastrin promoter alone and graphically plotted. (C) AGS cells were co-transfected with pGAS-Luc in combination with either pWTp65NFkB or pS536A p65 NFkB with and without pHDAC1 expression vector. Cells were harvested after 48 hr of transfection. The mean luciferase activity of three different experiments is represented as Relative Luciferase Unit/µg protein with respect to gastrin promoter alone and graphically plotted. (D) Co-immunoprecipitation assay confirms the association of NFkB p65 with either p300 or HDAC1 in IL1B stimulus specific manner. AGS cells were either treated with 10ng/ml of recombinant IL1B or left untreated for two hr. Lysates were prepared for immunoprecipitation with either p300 or HDAC1. Immunoprecipitated protein complexes were then immunoblotted with NFkB p65 antibody. (E) The band intensities of co-immunoprecipitation experiments were scanned by imageJ and their mean normalized density of three independent experiments with +/-SD values graphically plotted.

In a previous study we observed that IL1B-mediated gastrin repression was abrogated by the HDAC inhibitor TSA [6]. However, the molecular mechanism underlying this inhibition was not investigated. As HDACs are a major class of chromatin modifying proteins that influence NFkB transcription [26], we tested the possibility that HDAC proteins may be involved in the NFkB-mediated repression of gastrin transcription. Co-transfection of pHDAC1 and pGas-Luc in AGS cells resulted in dose dependent inhibition of gastrin promoter activity by HDAC1 protein (Figure 3A). Interestingly, both NFkB p65 and HDAC1 mediated gastrin promoter repression was significantly alleviated in the presence of TSA and NBD (NFkB inhibitor), respectively (Figure 3B). Moreover almost complete alleviation was observed when both TSA and NBD were present together (Figure 3B). These results suggest that the interaction between HDAC1 and NFkB p65 is required for the repression of gastrin expression by NFkB. To further confirm that the interaction between NFkB p65 and HDAC1 is necessary for gastrin repression, we compared the gastrin promoter activity in AGS cells co-transfected with pHDAC1 and either WTp65NFkB or the pS536Ap65NFkB mutant. Figure 3C clearly shows that the inhibitory activity of HDAC1 was significantly abrogated in the presence of the S536A mutant when compared to its association with wild type NFkB p65. It should be noted, however, that pS536Ap65NFkB did affect gastrin expression to a lesser extent. Finally, co-immunoprecipitation analysis demonstrates that HDAC1 physically interacts with NFkB p65 more efficiently in the presence of IL1B while there is reduced interaction between NFkB p65 and p300 under the same condition (Figure 3D and 3E).

### IL1B-dependent HDAC1 and NCoR recruitment at the gastrin promoter results in gastrin repression by histone modifications

**Figure 4:**
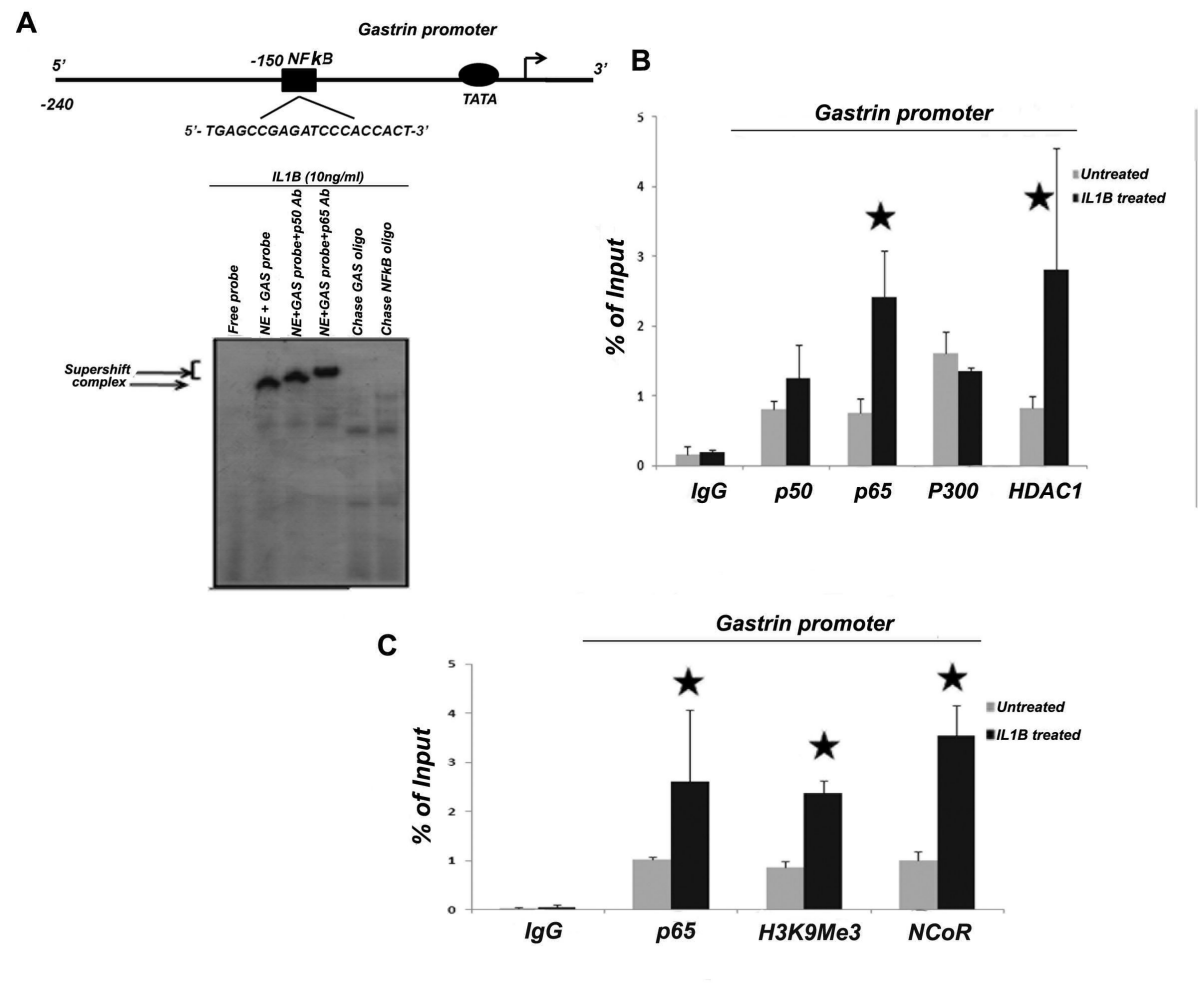
NFkB hetero-dimerize on the gastrin promoter and associate with either p300 or HDAC1 in a stimulus specific manner. (A) Direct binding of NFkB heterodimers to gastrin promoter. EMSA was performed with nuclear extracts prepared from IL1B treated (10ng/ml for 2hr.) AGS cells. Supershift assay was performed by incubating the nuclear lysates first with either NFkB p50 or p65 antibody for 30 min and then with radiolabelled gastrin EMSA probe. The chase was performed using corresponding unlabelled oligo probes. NE stands for Nuclear Extract. (B) Gastrin promoter occupancy by NFkB heterodimers and other co-factors. ChIP qPCR analysis was done with anti p50, p65, HDAC1 and p300 antibodies in IL1B (10ng/ml) treated and untreated AGS cells. (C) NFkB associates with HDAC1 and NCoR on the gastrin promoter to bring about histone methylation. ChIP qPCR analysis was done with anti NFkB p65, anti-H3K9Me3 and anti-NCoR in IL1B (10ng/ml) treated and untreated AGS cells.

**Figure 5:**
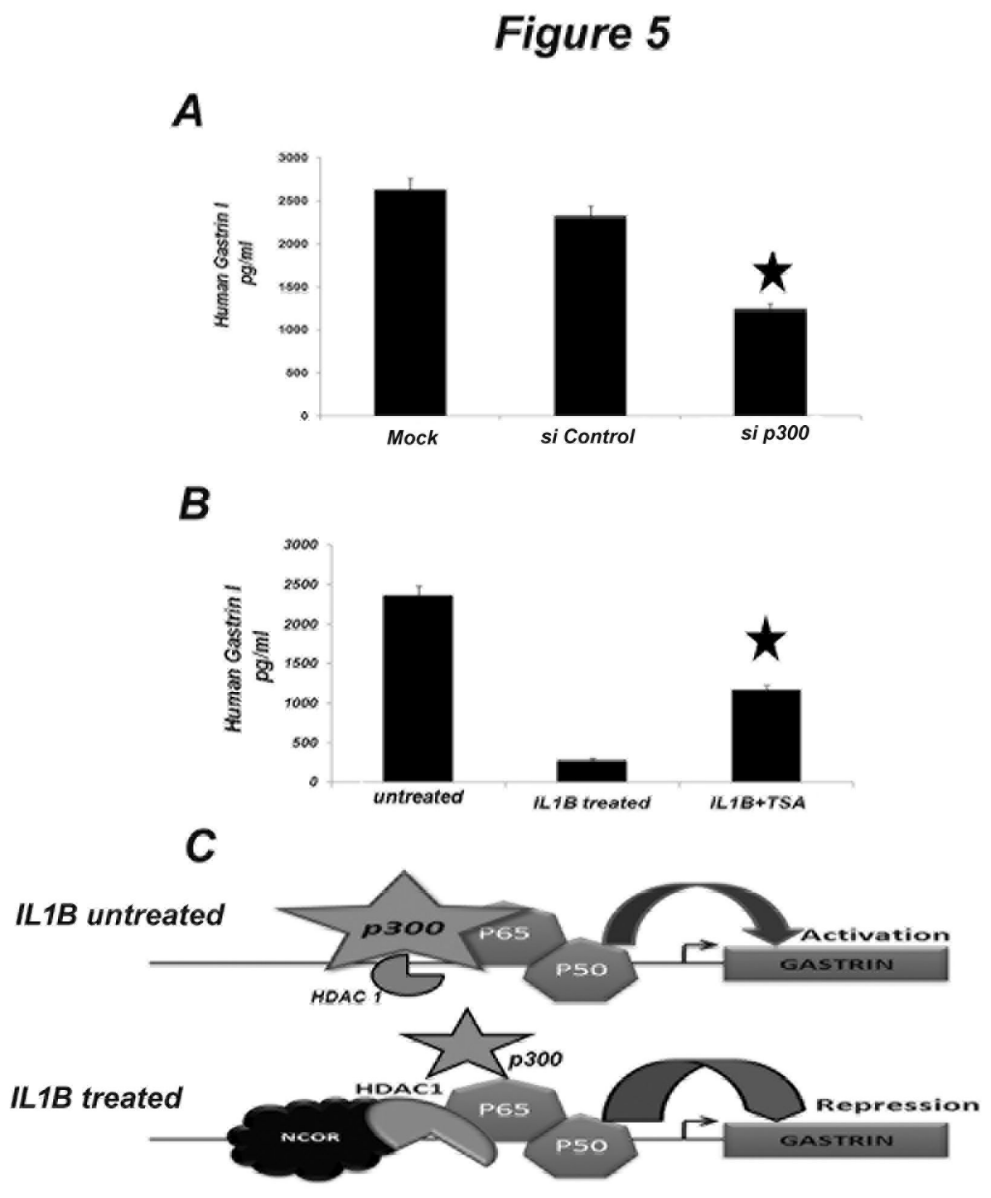
Modulation of p300 and HDAC1 alters endogenous gastin expression in AGS cells. (A) Knock down of p300 down regulates basal expression of endogenous gastrin. AGS cells were incubated with either scrambled control siRNA or with siRNA against p300 for 48 hr. Cell lysates were prepared and protein estimation was done. Equal amount of total protein was used for ELISA of Gastrin. (B) HDAC1 functionally attenuates gastrin expression. AGS cells were treated with either IL1B or IL1B along with TSA or left untreated. Gastrin ELISA was performed with equal amount of protein lysates prepared from these cells. (C) Gastrin expression is modulated by a stimulus specific alteration between NFkB-p300 and NFkB-HDAC1 recruitment (Upper panel). NFkB basally activates gastrin expression with p300 as a co-activator (Lower panel). In presence of IL1B there is an increased recruitment of HDAC1 and divestment of p300 that result in inhibition of gastrin transcription.

Binding of NFkB subunits on the gastrin promoter was validated by EMSA using probes designed either from the putative NFkB binding site on the gastrin promoter, or from the consensus NFkB site (Figure 4A and Figure S2A). Gastrin promoter occupancy of NFkB was further confirmed by a ChIP qPCR assay in AGS cells in the presence and absence of IL1B (Figure 4B). GAPDH promoter occupancy was monitored as a negative control in all ChIP qPCR experiments (Figures S2B and S2C). It may be reasoned that the presence of NFkB subunits on the gastrin promoter under the untreated condition can be attributed to basal gastrin expression. Next, we investigated the possible association of co-activators and co-repressors with NFkB on the gastrin promoter in the presence and absence of IL1B. We found that under the untreated condition, NFkB p65 co-immunoprecipitated with histone acetylase p300 (Figure 3D). The presence of p300 was also observed on the gastrin promoter along with NFkB subunits in un-stimulated cells (Figure 4B). In IL1B-stimulated cells, however, there was little change in the recruitment of p300 in contrast to significantly increased molecular abundance of HDAC1 on the gastrin promoter (Figure 4B). To further dissect the mechanism, we measured the endogenous gastrin-17 level under the following conditions: (a) AGS cells transfected with siRNA against p300 in the absence of IL1B and (b) AGS cells treated with HDAC inhibitor TSA in the presence of IL1B. We observed that knockdown of p300 resulted in decreased endogenous gastrin levels in AGS cells (Figure 5A), as expected. On the other hand, inhibition of HDAC1 activity by TSA led to the partial release of IL1B-mediated gastrin repression (Figure 5B). Figure S3 depicts that siRNA against p300 substantially lowered p300 mRNA expression in AGS cells (Figure S3). These results indicate that there is a change from the basal transcription activation mode (due to the presence of p300) to a repression mode upon stimulation by IL1B, which promotes the association of NFkB p65 with HDAC1 (Figure 5C). It is known that HDAC1 is involved in transcriptional repression by forming complexes with other repressor molecules such as mSin3 and NCoR [27]. Thus, the recruitment of these co-repressors on the gastrin promoter was investigated in the presence of IL1B by ChIP. Our results show that along with HDAC1, there was also recruitment of NCoR on the gastrin promoter in the presence of IL1B (Figure 4C). Recruitment of such repressor complex is followed by histone modifications. Methylation at the lysine 9 of histone 3 (H3K9Me3) is widely recognized as a signature of gene repression [20]. The gastrin promoter was checked for such signatures in AGS cells treated with recombinant IL1B (10ng/ml). Figure 4C shows the presence of methylated histone at the gastrin promoter in the presence of IL1B. These results suggest that IL1B recruits NFkB along with HDAC1 and NCoR to bring about gastrin repression via histone methylation.

## Discussion

In this study we have elucidated the signaling events that lead to IL1B-mediated repression of gastrin expression through NFkB activation in gastric carcinoma cells. We have investigated the mechanism by which the NFkB p65 subunit alters from activator mode to repressor mode in response to IL1B. IL1B-mediated NFkB activation might involve a number of MAP kinase pathways [28]. Antagonistic studies using pharmacological molecules indicated that IL1B does not transduce signal through the ERK, JNK, p38, and other protein kinase pathways to exert its effect on gastrin. On the contrary, this study delineated that IL1B-mediated down-regulation of gastrin is achieved by NFkB activation via the MyD88-TAK1 pathway (Figure 1G and [6]).

In cells, NFkB exists as a heterodimer comprised primarily of p50 and p65 protein subunits [8,9]. In the absence of activating signals, NFkB is sequestered in the cytoplasm by the inhibitor protein IkBα. Biological signals that induce phosphorylation of IkBα release the sequestered NFkB, allowing it to translocate to the nucleus and affect gene transcription [8,9,29]. Also, the p65 subunit of NFkB is known to be phosphorylated [30] by upstream kinases in response to a number of different stimuli, which may modulate the interaction of p65 with co-activator and co-repressor proteins [30]. In our study, the transcriptional potential of different mutant NFkB p65, with defects in the commonly known posttranslational modification sites, was investigated with respect to gastrin expression. The S536A phosphorylation mutant was shown to alleviate IL1B-mediated gastrin repression. Interestingly, we also observed that there was IL1B-induced phosphorylation of NFkB p65 at the S536 residue. It has been reported that phosphorylation at S536 increases the DNA binding ability of p65 and thus influence its transcriptional activity [19]. Though, to date, the S536 phosphorylation of NFkB p65 has been thought to be associated with transcriptional activation of the gene [31–34], our results indicate that IL1B-induced phosphorylation at S536 of NFkB p65 stabilizes the protein-DNA complex, and thereby helps sustain the inhibitory action of IL1B. We also observed that even in un-stimulated cells, the S536A mutant does not significantly repress gastrin compared to wild type NFkB p65. This observation emphasizes the importance of the S536 residue in the inhibition of gastrin expression, and suggests that the S536A mutant probably has lower DNA binding affinity than the wild type NFkB p65.

Gene transcription is a dynamic process governed by the extent and level of histone activity at a given promoter. Among the most studied histone modifications known to influence gene transcription is histone acetylation, which is governed by the balanced activities of histone acetylases (HATs) and deacetylases (HDACs) [35]. Class I HDACs, which include HDAC1, HDAC2, HDAC3, and HDAC8, are recruited to DNA as part of a large multiprotein repressor complex comprised of Sin3 or Mi-2-NuRD proteins [27]. One paradigm supports that HDACs inhibit gene transcription by deacetylating histone core proteins [26], which compacts the nucleosome, sterically impairing access of transcription factors to DNA [20,35]. In addition to histone modification, HDACs can also influence gene transcription by modifying non-histone DNA binding factors as well. Indeed, HDACs have been reported to impair gene transcription by interacting with certain transcription factors [36,37]. Likewise, in this study we show that the HDAC inhibitor TSA alleviates the inhibitory activity of NFkB p65 on gastrin gene transcription. Furthermore, over-expression of HDAC1 alone, as well as in the presence of wild type NFkB p65, resulted in increased inhibition of gastrin. Interestingly, there was alleviation of HDAC1-mediated repression in the presence of the NFkB inhibitor, NBD, which suggests that inhibition by HDAC1 is dependent on the presence of wild type NFkB p65. Indeed, co-immunoprecipiation confirmed the association of p65 with HDAC1 in IL1B-treated cells. Thus, these results suggest that the association of NFkB and HDAC1 brings about transcriptional repression of gastrin. Since over-expression of HDAC1 with S536Ap65NFkB did not repress gastrin expression as strongly as the case of the wild type, we conclude that the DNA binding ability of p65 is important in bringing about gene repression. Thus, our study highlights a less defined but emerging role for NFkB as a transcriptional repressor [38,39].

We observed the presence of NFkB heterodimers on the gastrin promoter both in IL1B stimulated and un-stimulated cells. The function of this nuclear NFkB in un-stimulated cells is not clear, although we presume that it may be involved in regulating basal gene expression. The reduced promoter activity upon deletion of the NFkB site from gastrin promoter led us to investigate a possible positive role of NFkB in basal gene transcription. Gene transcription is known to be regulated by transcription factors in association with various co-regulatory (co-activator and co-repressor) proteins [11]. These co-regulators function both by coupling sequence specific transcription factors with basal transcription machinery and altering chromatin structure via histone modifications [11]. A few important co-activator proteins include CREB-binding protein (CBP) and its structural homologue p300 [40], steroid receptor-coactivator-1 (SRC-1) [41,42], and p300/CBP associated factor (PCAF) [43]. Since NFkB is commonly known to associate with p300, we looked for its presence on the gastrin promoter. We observed that, in un-stimulated cells, p300 forms a complex with NFkB p65, and is also present on the promoter. Knockdown of p300 with siRNA in AGS cells resulted in lower basal gastrin expression. These observations strongly suggest that NFkB has a positive role in the basal transcription of gastrin. However, stimulating the cells with IL1B shifts the co- regulatory complex and recruits HDAC1 along with NCoR to gastrin promoter, which participates along with NFkB p65 to cause gastrin repression. Thus, this study reveals that the association of different sets of co-factors with NFkB converts the basal transcriptional activator into a repressor in the presence of an external stimulus like IL1B (Figure 5C). This observation is important as it explains the potential role of co-regulatory proteins in modulating the trans-activation potential of NFkB. Although it is reported by Zhong et al. [30] that phoshorylation at S276 of NFkB is crucial for its association with p300, we did not observe any significant modification on NFkB p65 other than S536 phosphorylation. Thus, the exact mechanism of IL1B-mediated change in transcriptional activity remains elusive. Further investigation is required to find out whether modifications at other amino acid residues of NFkB p65, yet largely unexplored, are involved in this process.

## Supporting Information

Figure S1 **Modulation of TAK1 and TAB1 expression in AGS cells.** (A) Ectopic expression of pCMVTAK1 and pCMVTAB1 in AGS cells. AGS cells were co-transfected in a dose dependent manner with pCMVTAK1 along with its activator pCMVTAB1 (0.2µg) and immunoblotted with anti TAK1 and TAB1 antibodies to check for the expression. A representative blot is shown. (B) siRNA-mediated knock down of TAK1 in AGS cells. Total RNA was extracted from AGS cells 48 hr after transfection with either TAK1 siRNA (80 nM) or control siRNA (80 nM). Real time PCR analysis for TAK1 expression was performed in those RNA samples. 18srRNA was taken as the endogenous control. The graph represents the mean of relative quantification measured from three different experiments +/- SD.
(TIF)

Figure S2 **Control experiments of EMSA and ChIP qPCR analysis.** (A) EMSA was performed using consensus NFkB binding site as the oligo probe and nuclear extracts of AGS cells treated with IL1B (10ng/ml) for 2 hr. Supershift assay was performed by incubating the nuclear lysates first with either NFkB p50 or p65 antibody for 30 min and then with radiolabelled NFkB consensus oligo. The chase was performed using corresponding unlabelled oligo probes. NE stands for Nuclear Extract. (B) ChIP qPCR was done with anti NFkB p50, p65, HDAC1 and p300 antibodies in IL1B (10 ng/ml) treated and untreated AGS cells using GAPDH promoter primers to check for their respective promoter occupancy. (C) ChIP qPCR was done with anti NFkB p65, anti H3K9Me3 and anti NCoR in IL1B (10ng/ml) treated and untreated AGS cells using GAPDH promoter primers to check for their respective promoter occupancy.
(TIF)

Figure S3 **siRNA-mediated knock down of p300 in AGS cells.** Total RNA was extracted from AGS cells after 48 hr of transfection with either p300 siRNA (80 nM) or control siRNA (80 nM). Real time PCR analysis for p300 expression was performed in those RNA samples. 18srRNA was taken as the endogenous control. The graph represents the mean of relative quantification measured from three different experiments +/- SD.
(TIF)
