# Supplementary figures and images for: NF-kappaB Mediated Transcriptional Repression of Acid Modifying Hormone Gastrin
Source: PLoS One. 2013 Aug 23;8(8):e73409. doi: 10.1371/journal.pone.0073409 (PMC3751843; doi:10.1371/journal.pone.0073409)

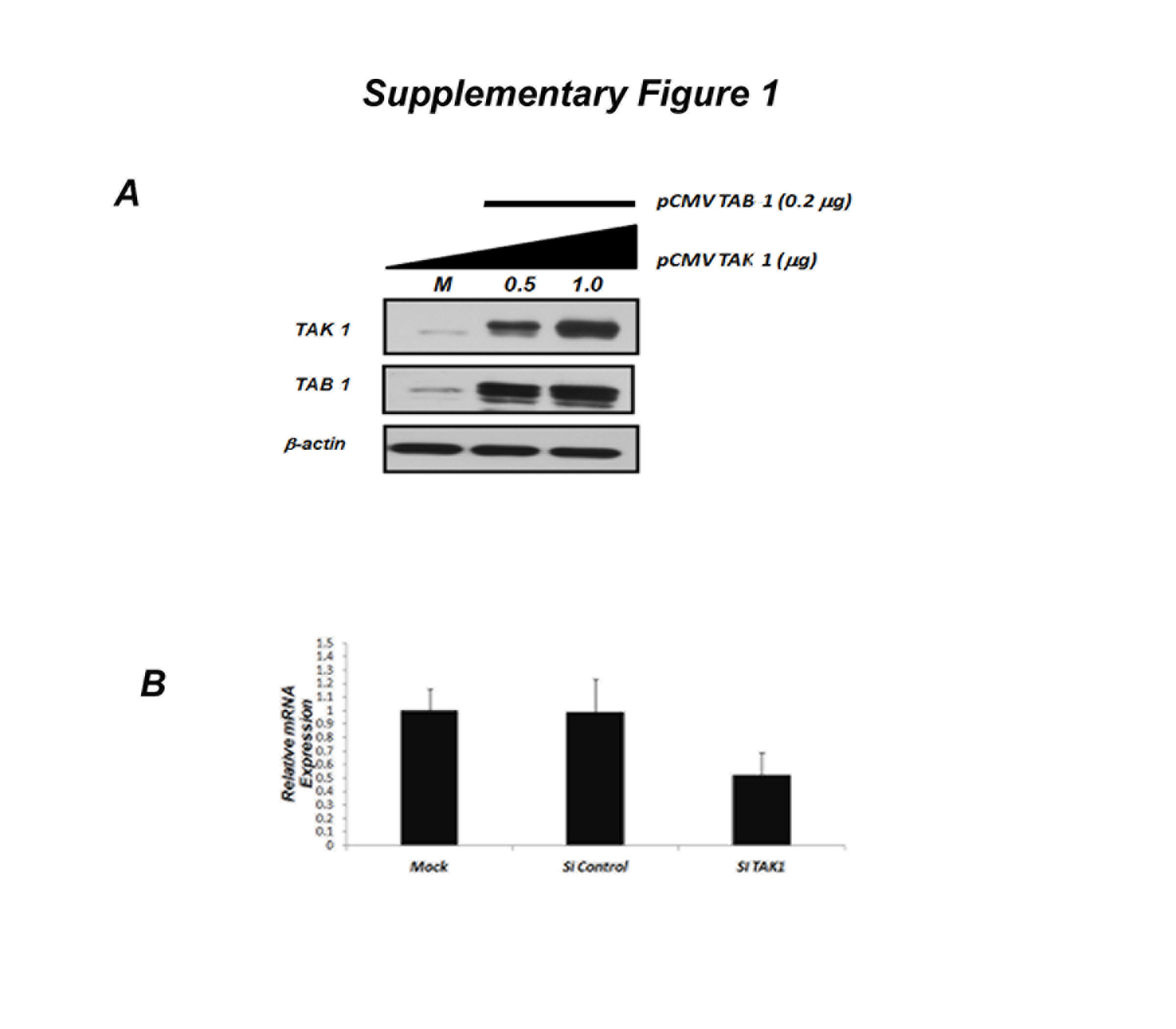

Supplement: Figure S1 — Modulation of TAK1 and TAB1 expression in AGS cells. (A) Ectopic expression of pCMVTAK1 and pCMVTAB1 in AGS cells. AGS cells were co-transfected in a dose dependent manner with pCMVTAK1 along with its activator pCMVTAB1 (0.2µg) and immunoblotted with anti TAK1 and TAB1 antibodies to check for the expression. A representative blot is shown. (B) siRNA-mediated knock down of TAK1 in AGS cells. Total RNA was extracted from AGS cells 48 hr after transfection with either TAK1 siRNA (80 nM) or control siRNA (80 nM). Real time PCR analysis for TAK1 expression was performed in those RNA samples. 18srRNA was taken as the endogenous control. The graph represents the mean of relative quantification measured from three different experiments +/- SD. (TIF) [file pone.0073409.s001.tif]

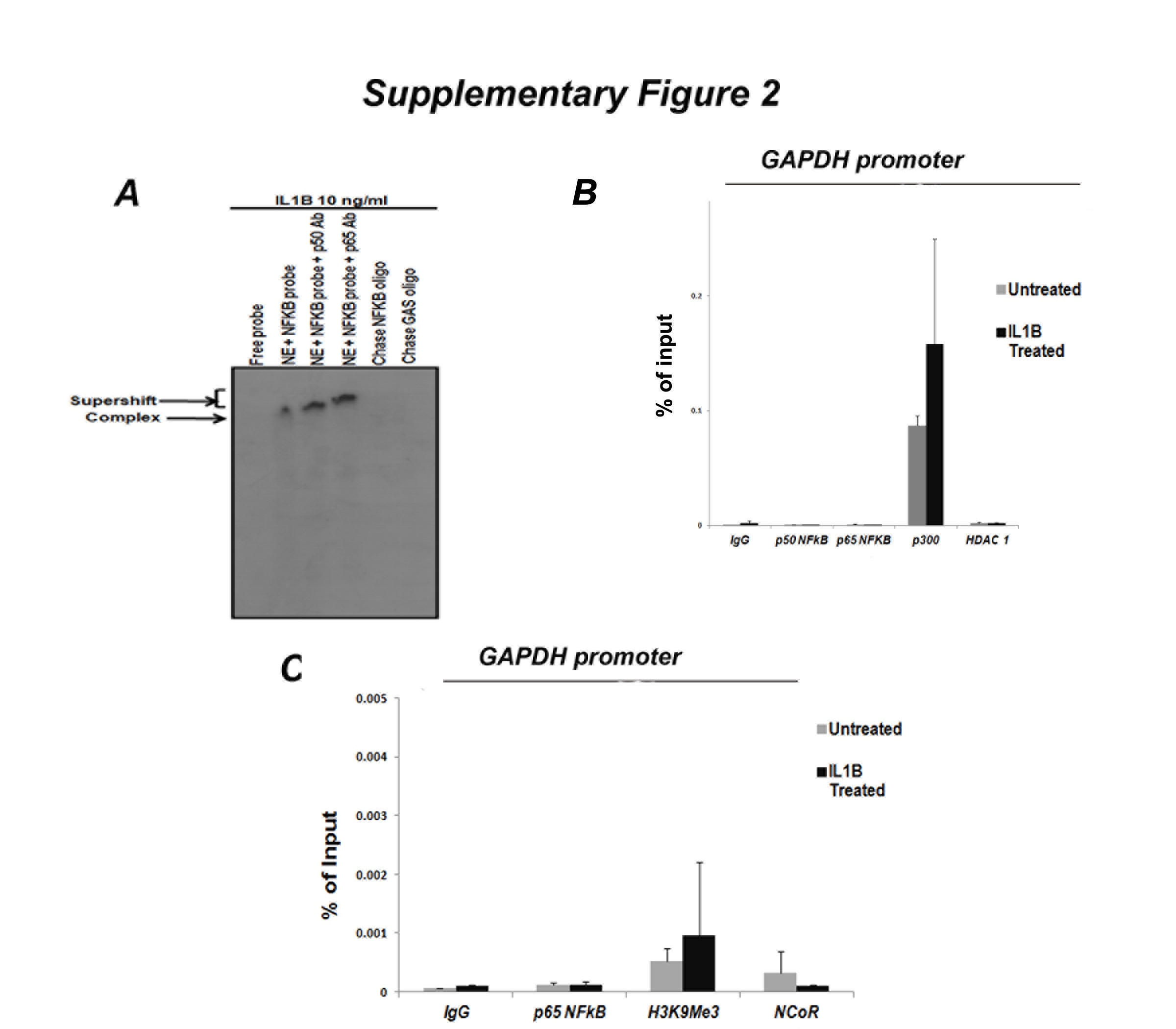

Supplement: Figure S2 — Control experiments of EMSA and ChIP qPCR analysis. (A) EMSA was performed using consensus NFkB binding site as the oligo probe and nuclear extracts of AGS cells treated with IL1B (10ng/ml) for 2 hr. Supershift assay was performed by incubating the nuclear lysates first with either NFkB p50 or p65 antibody for 30 min and then with radiolabelled NFkB consensus oligo. The chase was performed using corresponding unlabelled oligo probes. NE stands for Nuclear Extract. (B) ChIP qPCR was done with anti NFkB p50, p65, HDAC1 and p300 antibodies in IL1B (10 ng/ml) treated and untreated AGS cells using GAPDH promoter primers to check for their respective promoter occupancy. (C) ChIP qPCR was done with anti NFkB p65, anti H3K9Me3 and anti NCoR in IL1B (10ng/ml) treated and untreated AGS cells using GAPDH promoter primers to check for their respective promoter occupancy. (TIF) [file pone.0073409.s002.tif]

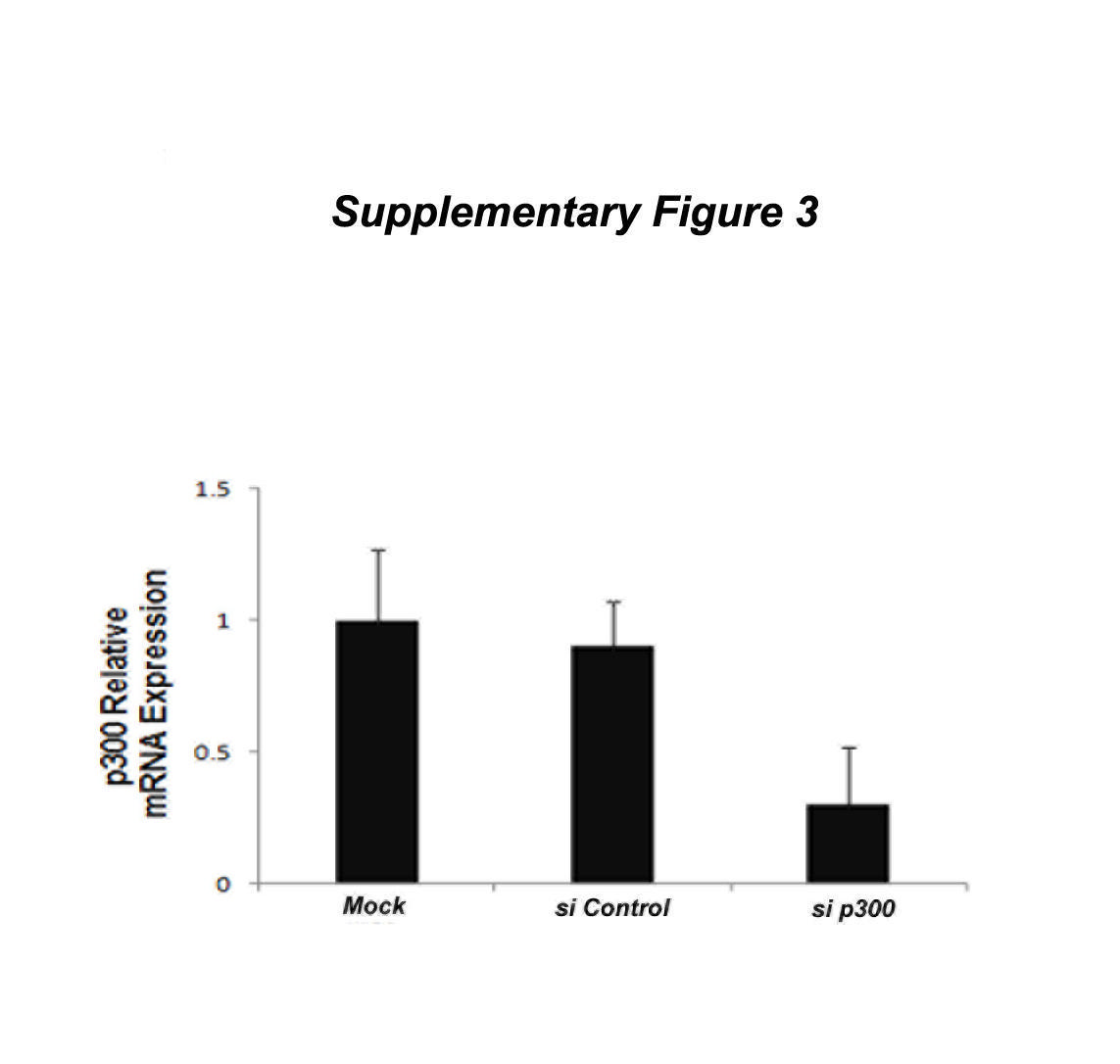

Supplement: Figure S3 — siRNA-mediated knock down of p300 in AGS cells. Total RNA was extracted from AGS cells after 48 hr of transfection with either p300 siRNA (80 nM) or control siRNA (80 nM). Real time PCR analysis for p300 expression was performed in those RNA samples. 18srRNA was taken as the endogenous control. The graph represents the mean of relative quantification measured from three different experiments +/- SD. (TIF) [file pone.0073409.s003.tif]
